# Oral Epithelial Dysplasia Information Needs Questionnaire (ODIN‐Q): Responsiveness and Insights Into Patient Education

**DOI:** 10.1111/jop.70160

**Published:** 2026-06-12

**Authors:** Waleed Alamoudi, Abdullah Alsoghier, Richeal Ni Riordain, Stefano Fedele, Stephen Porter

**Affiliations:** ^1^ Faculty of Dentistry King Abdulaziz University Jeddah Saudi Arabia; ^2^ UCL Eastman Dental Institute, University College London London UK; ^3^ College of Dentistry King Saud University Riyadh Saudi Arabia; ^4^ Cork University Dental School and Hospital, University College Cork Cork Ireland; ^5^ Oral Theme of the UCL/UCLH NIHR Biomedical Research Centre London UK

**Keywords:** informational needs, ODIN‐Q, oral epithelial dysplasia, patient education, patient‐reported outcome measures, psychometric properties, responsiveness

## Abstract

**Background:**

The Oral Epithelial Dysplasia Informational Needs Questionnaire (ODIN‐Q) was developed to assess the informational needs of patients with oral epithelial dysplasia (OED), a precancerous disorder associated with an increased risk of malignant transformation. This study aimed to evaluate the responsiveness of the ODIN‐Q following an educational intervention using a written patient information leaflet about OED.

**Methods:**

A prospective pre‐post observational study was conducted at the Oral Medicine Unit at University College London Hospitals, between March 2023 and March 2025. Fifty patients with histologically confirmed OED completed the previously validated ODIN‐Q before and after reading the leaflet. Differences between pre‐ and post‐reading ODIN‐Q scores were analysed using descriptive statistics and Cohen's *d* to determine effect sizes and responsiveness.

**Results:**

Fifty participants (29 females, 21 males; mean age = 65 years) were included in the analysis. Overall ODIN‐Q scores increased from 2.44 to 2.71, with a small overall effect size (*d* = 0.30; 95% CI: 0.01–0.59). The highest responsiveness was observed in the medical system and access to information domain (+0.42; +19%; *d* = 0.49; 95% CI: 0.19–0.79), followed by psychosocial aspects (+0.29; +12%; *d* = 0.38; 95% CI: 0.09–0.67) and physical aspects (+0.28; +11%; *d* = 0.36; 95% CI: 0.07–0.65). Other domains showed negligible to small responsiveness (investigative tests: *d* = 0.11; 95% CI: −0.18–0.40).

**Conclusions:**

The ODIN‐Q demonstrates preliminary evidence of responsiveness to changes in patients' informational needs following educational interventions, supporting its potential use as a multidomain measure for evaluating and guiding patient‐centred education in OED.

## 1 Introduction

Oral epithelial dysplasia (OED) is a histopathological diagnosis characterised by abnormal patterns of cellular maturation and proliferation. Although the mechanisms driving malignant transformation in OED remain unclear, it is well established that OED represents a precursor state with the potential to progress to oral squamous cell carcinoma [1]. Management strategies are patient‐specific and informed by histological grade of dysplasia, along with other clinical and patient factors; some individuals remain under long‐term surveillance, while others undergo surgical excision [2]. Consequently, individuals undergoing repeated healthcare visits for observation, investigations, and treatment for OED may experience physical, psychological, and emotional concerns, mainly stemming from fears about cancer development or recurrence [3].

Access to comprehensible health information can reduce patient anxiety and support their informed decision‐making [4]. Well‐informed individuals experience greater certainty, improved coping strategies, higher satisfaction, and likely better treatment outcomes [5]. Despite this, discrepancies often exist between patients' information needs (IN) and the information clinicians provide or consider relevant, which may hinder shared decision‐making and effective patient–clinician communication [6].

To address these gaps, the oral epithelial dysplasia informational needs questionnaire (ODIN‐Q) was developed in the UK as a comprehensive 33‐item instrument designed to assess the IN of patients with OED [7]. The tool covers multiple domains, including demographic and clinical information, disease knowledge, investigations, treatment, physical and psychological concerns, healthcare systems, and access to information resources. Its conceptual foundation draws on Lazarus' (1984) stress, appraisal, and coping theory, which posits that acquiring information and adopting proactive strategies can function as key coping mechanisms in the context of encountering stressful health‐related diagnoses [8].

Initial validation studies demonstrated that the ODIN‐Q possesses strong content and face validity, excellent internal consistency (Cronbach's *α* = 0.93), and moderate test–retest reliability (*κ* = 0.49–0.53) [7]. Convergent validity was established by comparison with an existing tool, aligning with recommended standards for construct validation [9]. Further construct validity was supported by hypothesis testing [9], which showed that patients with multiple comorbidities consistently reported greater unmet IN than those with fewer or no conditions. These findings reflect Lazarus' theoretical underpinnings that individuals confronting additional stressors, such as a new precancer diagnosis alongside other health concerns, are more inclined to seek further information as a coping strategy.

More recently, confirmatory factor analysis has provided further psychometric evidence, indicating that the six‐factor ODIN‐Q structure is both statistically and conceptually robust for evaluating patient informational needs in OED [10]. While minor revisions were suggested, the six‐domain model effectively captured the multidimensional nature of patient concerns, with average factor loadings of 0.58 (range, 0.28–0.84), and only two items fell below the 0.30 threshold [10]. Notably, the tool demonstrated conceptual distinctiveness, with low inter‐factor correlations (< 0.20).

Despite these strengths, further work is required to assess the responsiveness of the ODIN‐Q—its ability to detect changes in IN over time, particularly before and after educational interventions. Responsiveness is a critical property of outcome measures, enabling researchers and clinicians to evaluate the impact of tailored support and to monitor patients' evolving needs. Without validated and responsive instruments, reliance on untested tools risks generating inaccurate or misleading findings, which can undermine intervention planning and lead to less effective patient support. Accordingly, this study aims to assess the responsiveness of the ODIN‐Q and its capacity to capture changes following the introduction of a patient information leaflet (PIL) to patients with OED.

## 2 Materials and Methods

### 2.1 Study Design and Participants

This prospective pre‐post observational study was conducted as part of the patient education in OED (EDUCAT‐ED) project, which aimed to identify the IN of patients with OED and develop educational resources tailored to those needs and preferences. The study was conducted between March 2023 and March 2025 at the Oral Medicine Unit of the Royal National ENT and Eastman Dental Hospitals, University College London Hospitals (UCLH).

Previously, Phase I of EDUCAT‐Ed included 165 patients with OED who were recruited through convenience sampling to complete the ODIN‐Q, aiming to evaluate disease‐specific IN [11], and conduct confirmatory factor analysis [10]. Phase II focused on assessing responsiveness, and 50 participants who had already taken part in Phase I were invited to complete the ODIN‐Q again. These 50 participants were a convenience subsample of the 165 Phase I participants, selected based on availability and willingness at the time of PIL distribution. No formal comparison was conducted between participants and non‐participants. In line with the COSMIN guidelines, a sample size of 50 meets the threshold for an ‘adequate’ rating for responsiveness studies [9].

Participants were eligible if they were aged 18 years or older, diagnosed with OED according to the 2017 World Health Organization diagnostic criteria, resident in the UK, proficient in spoken and written English, able to provide informed consent, and not undergoing radiotherapy or chemotherapy for head, neck, or other cancers. All diagnoses were histologically confirmed, either at UCLH or external facilities. Data were collected only during the follow‐up phase of care, meaning no participants were enrolled before biopsy or after discharge.

Eligible patients received a verbal explanation of the study, followed by a study information sheet and were asked to provide written consent. They were then given a printed copy of the ODIN‐Q to complete on two occasions: once before receiving the PIL, either during their clinic visit or at home, and again immediately after reading the PIL about OED. Participants who completed the post‐reading ODIN‐Q at the clinic visit returned it in person on the same day; those who completed it at home were asked to return it by post within 7–14 days.

### 2.2 Measurements

#### 2.2.1 Oral Epithelial Dysplasia Information Needs Questionnaire

The ODIN‐Q is structured into three sections (Table 1). The first section gathers socio‐demographic information along with details on smoking and alcohol use. The second section contains 33 items that assess the sufficiency of the information provided about OED, rated on a four‐point scale (1 = none, 2 = not enough, 3 = enough, 4 = too much). Scores range from 33 to 132, with higher values indicating greater adequacy of information. The third section explores patients' preferences for how they would like to receive information.

**TABLE 1:** Content and response choices of the ODIN‐Q.

ODIN‐Q section	Number of items	Components	Response choices
Socio‐demographics	7	Age, race, ethnicity, level of education, employment status, and smoking and alcohol intake	Open‐ended, closed‐ended, multiple‐choice
Level of information received	33	Six categories, involving questions on knowledge about the disease, investigative procedures, treatments, physical and psychosocial aspects, and medical access and information availability	0 = not applicable
			1 = not at all
			2 = not enough
			3 = enough
			4 = too much
Preferred methods of information delivery	1	Individual meetings, printed materials, audiovisual resources, and group information sessions.	Multiple‐choice

Abbreviation: ODIN‐Q, oral epithelial dysplasia informational needs questionnaire.

#### 2.2.2 Patient Information Leaflet

**FIGURE 1:**
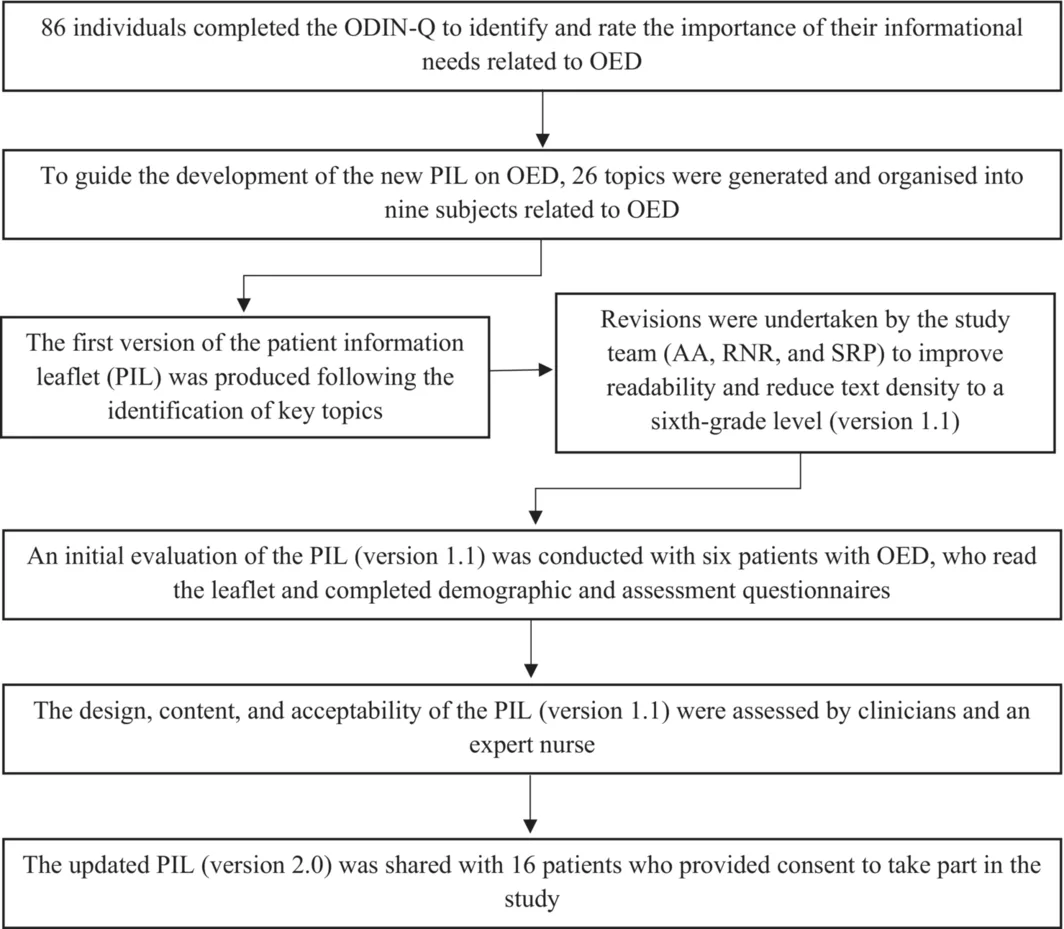
Overview of the development and preliminary testing process for the patient information leaflet.

As part of the ODIN‐Q project [7], a written information material was developed at the oral medicine unit of UCLH. The processes involved in the development and preliminary testing of the new PIL during the ODIN‐Q project are summarised in Figure 1.

### 2.3 Ethical Considerations and Study Registration

The study was conducted in strict accordance with the ethical principles outlined in the 2024 WMA Declaration of Helsinki—Ethical Principles for Medical Research Involving Human Participants (World Medical Association. World Medical Association Declaration of Helsinki: Ethical Principles for Medical Research Involving Human Participants. *JAMA*. 2025;333(1):71–74. doi: https://doi.org/10.1001/jama.2024.21972). The protocol underwent independent expert review to ensure both scientific rigour and feasibility. The study was formally registered with the Joint Research Office of UCLH and UCL [reference number (EDGE number): 153912 and Integrated Research Application System project ID: 318039]. Ethical approval was granted on 11 January 2023 by the London–Surrey Borders Research Ethics Committee [reference 22/PR/1743] and the NHS‐England Health Research Authority.

### 2.4 Analysis of Data and Responsiveness

Data were analysed and represented using descriptive statistics to summarise demographic and clinical characteristics. Responsiveness was evaluated by comparing ODIN‐Q scores before and after administration of the PIL, using the scoring framework described in Table 1. The magnitude of change was expressed as Cohen's *d*, a standardised effect size that reflects the difference in scores relative to baseline variability. Effect sizes were interpreted according to established thresholds: negligible (< 0.2), small (0.2–0.49), medium (0.5–0.79), and large (≥ 0.8). In this analysis, higher mean scores following the intervention indicated a reduction in patients' perceived IN, suggesting a beneficial impact of the educational leaflet.

## 3 Results

### 3.1 Socio‐Demographic Characteristics of Participants

Table 2 summarises the socio‐demographic and clinical characteristics of the study cohort (*n* = 50). The participants consisted of 29 females (58%) and 21 males (42%), with ages ranging from 35 to 80 years, and a mean and median age of 65 years. Seven participants (14%) were current smokers, 23 (46%) reported past use of tobacco (cigarettes or smokeless forms), and 20 (40%) had never smoked. Twenty‐six participants (52%) reported current alcohol consumption, 11 (22%) reported past use, and 13 (26%) had never consumed alcohol.

**TABLE 2:** Socio‐demographic and clinical characteristics of participants (*n* = 50).

Variable	Category	Number (%)
Sex	Female	29 (58.0%)
	Male	21 (42.0%)
Age (years)	30–39	1 (2.0%)
	40–49	3 (6.0%)
	50–59	9 (18.0%)
	60–69	21 (42.0%)
	70–79	14 (28.0%)
	80–89	2 (4.0%)
Ethnicity	White (British)	26 (52.0%)
	White (other)	11 (22.0%)
	Asian or Asian British	12 (24.0%)
	Black or Black British	1 (2.0%)
Education	College or higher educational degree	31 (62.0%)
	High school diploma or less	18 (36.0%)
	Not reported	1 (2.0%)
Employment	Retired	26 (52.0%)
	Employed (full‐time)	10 (20.0%)
	Employed (part‐time)	3 (6.0%)
	Self‐employed	6 (12.0%)
	Unemployed	3 (6.0%)
	Not reported	2 (4.0%)
Tobacco use (cigarettes and smokeless)	Current	7 (14.0%)
	Past	23 (46.0%)
	Never	20 (40.0%)
Alcohol consumption	Current	26 (52.0%)
	Past	11 (22.0%)
	Never	13 (26.0%)
OED histopathological examination	Mild dysplasia	44 (51.2%)
	Moderate dysplasia	30 (34.9%)
	Severe dysplasia	12 (14.0%)
OED sites	Tongue	27 (54.0%)
	Buccal mucosa	14 (28.0%)
	Gingiva	10 (20.0%)
	Hard palate	4 (8.0%)
	Floor of the mouth	4 (8.0%)
	Soft palate	1 (2.0%)
Associated oral disease	Oral lichen planus	32 (64.0%)
	Oral leukoplakia	11 (22.0%)
	Oral submucous fibrosis	3 (6.0%)
	Oral candidiasis	1 (2.0%)

Abbreviation: OED, oral epithelial dysplasia.

Histopathological reports identified a total of 86 dysplastic lesions. Mild dysplasia was the most common diagnosis (*n* = 44; 51.2%), followed by moderate (*n* = 30; 34.9%) and severe (*n* = 12; 13.9%). Across the cohort, 61 oral mucosal sites were recorded, as some participants presented with lesions that extended to or developed at multiple sites. The tongue was the most frequently affected (*n* = 27; 54%), followed by the buccal mucosa (*n* = 14; 28%) and the gingiva (*n* = 10; 20%), with other sites less commonly involved. In addition, 32 participants (64%) presented with clinical and/or histological diagnosis of oral lichen planus (OLP), 11 (22%) had oral leukoplakia, three (6%) had oral submucous fibrosis, and only one (2%) had oral candidiasis (Table 2).

### 3.2 Responsiveness

Fifty participants from the original cohort were provided with the PIL and asked to complete the ODIN‐Q to assess potential changes in their responses over time. Overall, ODIN‐Q scores increased from 2.44 to 2.71, representing an average improvement of 0.27 points (≈11%) and a small overall effect size (*d* = 0.30; 95% CI: 0.01–0.59). The highest responsiveness occurred in the medical system and access to information domain (*d* = 0.49; 95% CI: 0.19–0.79), followed by psychosocial aspects (*d* = 0.38; 95% CI: 0.09–0.67) and physical aspects (*d* = 0.36; 95% CI: 0.07–0.65). Tables 3a and 3b presents the baseline and post‐intervention mean scores for each domain of the ODIN‐Q, along with the calculated effect sizes (Cohen's *d*), 95% confidence intervals, and interpretation of responsiveness.

**TABLE 3a:** Mean ODIN‐Q scores and responsiveness by domain following the educational intervention.

Domain	Baseline mean	Follow‐up mean	Mean Δ^a^	% Change^a^	Mean Cohen's *d* (95% CI)^b^	Interpretation
Information about the disease	2.58	2.72	+0.14	+5.4%	0.18 [−0.11, 0.47]	Negligible–Small
Information about investigative tests	2.86	2.91	+0.05	+1.7%	0.11 [−0.18, 0.40]	Negligible
Information about treatment	2.45	2.66	+0.21	+8.6%	0.29 [−0.00, 0.58]	Small
Physical aspects	2.46	2.74	+0.28	+11.4%	0.36 [0.07, 0.65]	Small‐medium
Psychosocial aspects	2.44	2.73	+0.29	+11.9%	0.38 [0.09, 0.67]	Small‐medium
Medical system and access to information	2.20	2.62	+0.42	+19.1%	0.49 [0.19, 0.79]	Small‐medium
Overall (ODIN‐Q total)	2.44	2.71	+0.27	+11.1%	0.30 [0.01, 0.59]	Small responsiveness

^a^ Mean Δ indicates the average change from baseline to follow‐up. Percentage change = $\left(\text{follow}-\mathrm{up}–\text{baseline}\right)/\text{baseline}\times 100$.

^b^ Cohen's *d* thresholds: small = 0.2, medium = 0.5, large = 0.8. Higher scores indicate greater perceived understanding or information satisfaction. 95% confidence intervals (shown in brackets).

**TABLE 3b:** Summary of ODIN‐Q domain interpretations following the educational intervention.

Domain	Interpretation
Disease information	Negligible‐to‐small responsiveness, with modest gains in understanding of HPV, disease grading, and progression.
Investigative tests	Minimal change, indicating a continued need for clearer information about screening and diagnostic procedures.
Treatment	Small responsiveness overall, reflecting improved understanding of treatment consequences and cure expectations, with moderate gains for complementary medicine.
Physical aspects	Small responsiveness in awareness of symptoms and nutritional impact, with moderate gains regarding understanding of disease spread.
Psychosocial aspects	Small responsiveness in coping perception and fear of progression, with moderate improvement in understanding of social effects.
Medical system and access	Highest responsiveness among domains, showing marked improvement in awareness of available support resources, professional communication, and health‐promotion opportunities.

## 4 Discussion

The ODIN‐Q demonstrated small but consistent responsiveness, with most items showing negligible to small effects and a subset reaching the moderate range. The highest proportional gain occurred in the medical system and access to information domain, highlighting improved awareness of available support, communication pathways, and health‐promotion opportunities. Although the overall effect size was small—a magnitude typical of single‐session or low‐intensity educational interventions—such changes can still carry clinical value by increasing perceived understanding, enabling more effective questions during consultations, and priming patients for shared decision‐making [12]. This instrument, by ascertaining domain‐specific comprehension gaps, may help clinicians tailor discussions and materials to each patient's needs. The observed changes—albeit modest—are consistent and point to the tool's potential for use in routine patient‐centred communication. Similar domain‐based tools, such as the Patient Concern Inventory for head and neck cancer [13], have demonstrated the value of structured, patient‐reported outcome measures in guiding personalised care in oncology‐adjacent settings, further supporting the clinical utility of the ODIN‐Q approach. It must be acknowledged, however, that the absence of a comparator group means that observed changes in ODIN‐Q scores cannot be solely attributed to the PIL. Alternative explanations—including testing effects, concurrent clinical counselling, and time‐related influences—cannot be excluded. The primary aim of this study was, nonetheless, to evaluate the responsiveness of the ODIN‐Q as a psychometric instrument—its capacity to detect changes in informational needs over time—rather than to establish the causal efficacy of the PIL as an intervention.

These findings extend a growing body of evidence on the responsiveness of patient‐reported outcome measures following brief educational interventions. The need for robust psychometric evaluation of patient informational needs instruments—with particular emphasis on responsiveness—has been highlighted [14]. Related constructs have demonstrated measurable responsiveness in other healthcare settings; for example, the Family Reported Outcome Measure has been evaluated for responsiveness and minimal important change [15]. This supports the value of establishing responsiveness as a core psychometric property of patient‐reported outcome measures used in clinical and educational contexts.

Evidence from oral health education similarly indicates that brief, well‐targeted interventions consistently yield small to moderate improvements in knowledge, attitudes, and behaviours across diverse populations and delivery modalities [16, 17, 18, 19, 20, 21, 22], with greater effects when reinforcement or repeated exposure is incorporated.

Our findings therefore fit a broader pattern: a tailored, accessible written resource produced measurable—albeit modest—reductions in patients' IN, with the strongest improvements in care system navigation and support access. Its impact on recognising investigative tests remained limited, an asymmetry that points directly to content refinement priorities—including more precise explanations of the screening rationale, testing details, and communication of results—for the next iteration of OED patient information.

### 4.1 Future Directions

The findings of this study provide a foundation for practical applications and future research in OED patient education. The immediate next step is the rollout of an information protocol within England, which could (1) develop concise, ODIN‐Q‐informed materials—including short videos and written information—covering risk factors, disease prognosis and surveillance, psychosocial impacts, and routes to clinical and community support; (2) integrate this information into routine care consultations using teach‐back methods, electronic patient portals, QR codes, and websites affiliated with non‐profit professional organisations; and (3) evaluate patient comprehension by collecting feedback on the clarity and actionability of the materials and measuring their impact on decision‐making and psychosocial outcomes.

Future work may also consider integrating established behavioural science frameworks—including locus of control theory [23], the Health Belief Model [24], and the Capability, Opportunity, Motivation–Behaviour model [25]—to maximise the effectiveness of educational tools by targeting patients' perceived susceptibility, barriers to care, and motivation to maintain health‐protective behaviours.

Beyond England, future research should seek to validate the ODIN‐Q across multiple centres and diverse healthcare settings, as multi‐site studies would provide important evidence on its cross‐cultural applicability. Conducting validation studies across Europe, the Americas, or Asia, for example, may provide insights into how cultural beliefs, family involvement, economic factors, and healthcare system differences shape patient concerns and needs [26]. Comparative work in regions with high prevalence of tobacco and betel quid use, such as India, will be essential in tailoring educational strategies for at‐risk populations.

In terms of patient population, future studies should develop condition‐specific educational materials that address the distinct informational needs of patients with co‐existing OED and OLP or other potentially malignant disorders.

Finally, further longitudinal studies are needed to capture how informational needs evolve throughout the care pathway—from diagnosis through treatment and follow‐up—and how different educational modalities such as written leaflets, video‐based content, and face‐to‐face counselling influence patient understanding over time. Embedding the ODIN‐Q into clinical practice as a decision‐support tool will help clinicians personalise education, improve shared decision‐making, and ultimately contribute to better patient outcomes in OED.

This study has some limitations. First, the sample was recruited from a single dental hospital, which limits the generalisability of findings to broader populations, as variations in health literacy, cultural background, and access to healthcare may influence how patients perceive and report their informational needs. Second, 64% of participants carried a concurrent diagnosis of OLP—a distinct condition with its own informational requirements. Since the educational intervention was OED‐specific, the PIL is unlikely to have adequately addressed the informational needs of patients managing both conditions simultaneously. Third, the time elapsed between the first and second ODIN‐Q completion was not recorded, and the interval between reading the PIL and completing the post‐reading ODIN‐Q varied among participants. Some individuals may have attended further clinic appointments or accessed additional health information in the intervening period, potentially influencing their post‐reading scores. Fourth, the ODIN‐Q has been reported to have moderate test–retest reliability (*κ* = 0.49–0.53) [7], and some of the observed pre‐and post‐reading change may reflect measurement error rather than a true shift in informational needs; however, the consistent direction of change across domains supports the true effect of at least part of the improvement. Fifth, the absence of a formal comparison between participants and non‐participants introduces a potential risk of selection bias. Finally, participant responses may have been influenced by social desirability bias, particularly in items related to knowledge or behaviour, potentially underestimating the actual within‐group differences.

In conclusion, the ODIN‐Q demonstrates preliminary evidence of responsiveness to changes in patients' informational needs following a brief educational intervention. The structured, multi‐domain format supports its potential application in both clinical and research contexts to evaluate and guide patient‐centred education in OED.

## Author Contributions

**Waleed Alamoudi:** writing – original draft, writing – review and editing, conceptualisation, methodology, funding acquisition, project administration, resources, investigation, formal analysis, data curation, and visualisation. **Abdullah Alsoghier:** writing – review and editing, conceptualisation. **Richeal Ni Riordain:** writing – review and editing, conceptualisation, methodology, funding acquisition, resources, supervision. **Stefano Fedele:** funding acquisition, project administration, resources, supervision. **Stephen Porter:** writing – review and editing, conceptualisation, methodology, funding acquisition, project administration, resources, investigation, supervision.

## Funding

There was no specific funding received for the present study from agencies in public, commercial, or not‐for‐profit sectors. W.A. held a PhD scholarship funded by King Abdulaziz University. S.P. received funding from the NIHR.

## Ethics Statement

Following registration with the University College London Hospitals/University College London Joint Research Office [reference number/EDGE number: 153912; IRAS project ID: 318039]. This study received a favourable opinion on 16 January 2023, from the NHS‐England's Research Ethics Committee [specifically, the London—Surrey Borders Research Ethics Committee, reference 22/PR/1743] and ethical approval was obtained on 26 January 2023 from the Health Research Authority and Health and Care Research Wales.

## Consent

Written informed consent was obtained from participants.

## Conflicts of Interest

The authors declare no conflicts of interest.

## Data Availability

The data that support the findings of this study are available on request from the corresponding author. The data are not publicly available due to privacy or ethical restrictions.
